# Rapid and Accurate ED-XRF Quantification of Trace Arsenic in Rice-Based Foods Employing ANNs to Resolve Lead Spectral Interference

**DOI:** 10.3390/foods15071130

**Published:** 2026-03-25

**Authors:** Murphy Carroll, Zili Gao, Lili He

**Affiliations:** Department of Food Science, University of Massachusetts Amherst, 102 Holdsworth Way, Amherst, MA 01003, USA; mmcarroll@umass.edu (M.C.); zgao@umass.edu (Z.G.)

**Keywords:** artificial neural network, regression, arsenic, lead, X-ray fluorescence spectrometry, quantification, food safety

## Abstract

Trace quantifications of arsenic (As) in foods by energy-dispersive X-ray fluorescence (ED-XRF) spectrometry are hindered by spectral overlap from lead (Pb) at characteristic emission lines. This study employed artificial neural networks (ANN) to statistically model and correct for As/Pb spectral overlap, enabling accurate As quantifications in rice-based foods. Calibration standards were prepared by pelletizing milled rice spiked with As and Pb, and validation was performed using a certified reference material, commercial rice-based foods, and Pb-spiked commercial foods. As calibration metrics were great (R^2^ = 0.92, standard error in calibration = 41.20 µg kg^−1^). The validation assessment achieved acceptable error using the As reference material (−19.43% error) and in commercial rice-based foods containing low Pb content (6 of 11 As determinations in agreement with the reference method). Additionally, accurate predictions of As were found in the presence of significant Pb interference (absolute mean error = 14.11% in Pb-spiked commercial foods). Overall, ANN modeling for Pb exhibited poor performance during both calibration and validation. This work demonstrates the usability of an ANN to address the As/Pb overlapping issue while offering insights into the strengths and weaknesses of ANNs when coupled with ED-XRF for trace elemental quantifications in foods.

## 1. Introduction

Arsenic (As) is listed as the top substance present in the environment which poses a threat to human health due to its toxic effects along with its frequency of exposure to humans [1,2]. One common route of oral exposure to As in humans is through foods, of which rice and rice-based foods are a common source of trace contamination and are recognized by many regulatory bodies high-risk foods [3,4,5,6,7]. Due to the high contamination risk, many regulatory bodies have recently updated or released guidelines outlining the maximum levels of As allowed in these foods. Many regulations have established upper limits at 200 µg kg^−1^ inorganic As (iAs) or below in rice and rice-based foods, with a specific focus on iAs species due to their heightened toxicity compared to their organic counterparts. Additionally, most regulatory documents specify maximum allowable limits of As in foods intended for infants and small children due to their increased susceptibility to the toxic effects of heavy metals compared to grown adults [4,5,6,7,8,9,10]. As the awareness of the risk of As contamination in these foods as well as the amount of regulation increases, there exists a heightened need for rapid analytical methods to screen total As content, enabling high-throughput preliminary analysis to reduce the risk of heavy metal exposure through food intake in infants and small children.

Energy dispersive X-ray fluorescence (ED-XRF) spectrometry is an atomic spectrometric tool that can be employed for the trace determination of heavy metals in foods. This technique enables the detection of a wide array of elements in a given sample due to the detection of characteristic fluorescent X-ray emissions [11]. In ED-XRF, high-energy incident photons are emitted onto a sample that displaces inner shell electrons, allowing for outer shell electrons to fill the vacancy in which the event releases fluorescent X-ray photons [11]. These characteristic emissions, dependent on the specific energies required to displace electrons of an element with a given atomic number (Z), are then detected and counted by a detector in the instrument [12]. Due to the characteristic nature of these emission lines for different elements, ED-XRF allows for the simultaneous quantitative determination of a wide range of elements, commonly from sodium to americium [13,14]. ED-XRF has been increasingly used for trace quantifications of heavy metals in a wide array of matrices [15,16,17,18,19] due to its rapid, simple, low-cost, and minimally destructive nature which boasts many advantages over traditional methods employed for elemental detection [13,20,21]. However, trace elemental quantification requires sufficient signal-to-noise ratios (SNRs) and spectral interferences to be absent or resolved through computational modeling in order to achieve reliable results. Consequently, due to an observed spectral overlap from Pb at its most intense spectral emission line, trace determinations of As by ED-XRF pose a significant challenge.

As and Pb both yield characteristic principal emission lines at around 10.5 keV, making quantifications of As difficult at trace concentrations when signals are diminished. The principal emission lines of As (Kα = 10.52 keV [22]) and Pb (Lα = 10.50 keV [22]) lie so close to one another that differentiating the elements based on these emission lines is impractical, as the resolution of most modern benchtop ED-XRF spectrometers is greater than 0.10 keV at Mn Kα [13,15,16,19]. To aid in the correction of spectral overlap at their principal lines, secondary lines resulting from the same ionization event (As Kβ = 11.73 keV, Pb Lβ = 12.62 keV) can be used; however, excitation probabilities of the β lines are much lower compared to the α lines for a given ionization event [11,23]. This results in much weaker SNRs and hinders the usability of β lines when employing an ED-XRF spectrometer to quantify As and Pb content near the detection limit of the instrument.

To improve quantifications of trace elemental concentrations when signals are diminished and noisy, chemometric data modeling systems can be employed. Chemometrics employ mathematical models to describe how a chemical or physical property of a sample is related to an instrumental signal [24], enabling the user to extract meaningful information from weak or overlapping signals and enhancing the ability to predict analyte concentrations from resultant spectra [25,26,27]. Chemometrics are often used for quantitative purposes with applications in ED-XRF spectrometry. Traditional linear approaches, such as simple linear regression, multiple linear regression, and partial least squares regression (PLSR) have been previously applied to ED-XRF to enable accurate elemental quantifications and correct for spectral overlap in a wide array of matrices [28,29,30,31]. These model types assume rigid linearity in the relationship between analyte concentration and instrumental response which may break down under conditions where spectral interferences are significant and SNR is weak, dominated by noise and matrix-dependent background signal. Therefore, recently the use of nonlinear regression models employing artificial neural networks (ANNs) has been increasingly explored. In many cases, these models are favored due to their flexibility and ability to model complex and nonlinear relationships, resulting in more robust predictions models compared to traditional techniques [26,32,33,34,35,36,37] when paired with sufficiently large training data sets and overfitting mitigation strategies [38]. ANN models measure weights between neurons containing three processing units—the input, output, and hidden units—to relate spectra to known concentrations with minimal error. Through iterative training, ANNs learn to model complex relationships between these two variables which traditional techniques may not be able to relate [35].

The objective of this study was to develop a method employing ED-XRF spectrometry coupled with appropriate sample preparation and data modeling techniques to correct for an observed spectral overlap between As and Pb, enabling accurate quantifications of As at trace levels in rice and rice-based foods. Calibration standards spiked with As and Pb were prepared, analyzed via an ED-XRF spectrometer, and the resultant spectra were used to generate calibration models utilizing an ANN. Limits of detection (LOD) and quantification (LOQ) were experimentally determined in two analytical scenarios in which samples contained varying amounts of As-Pb. The method was then validated using a certified reference material, commercial rice and rice-based foods, and commercial foods spiked with Pb. Predictions made by ANN modeling were compared to quantifications obtained via inductively coupled plasma-mass spectrometry (ICP-MS). The development of this method will enable accurate determinations of trace As content in rice and rice-based foods via ED-XRF spectrometry, resulting in a rapid, low-cost, and minimally destructive method allowing for heavy metal screening at levels aligning with current regulatory guidelines. Also, the assessment of model performance at varying As-Pb concentrations and investigation into the method’s application for Pb detection will highlight the strengths and weaknesses of ANNs, offering insight into their usability in specific analytical tasks.

## 2. Materials and Methods

### 2.1. Samples and Reagents

Dimethylarsinic acid (DMA) and lead (II) acetate trihydrate were obtained from Sigma-Aldrich (St. Louis, MO, USA). Enriched Calrose Medium Grain Rice used in development of calibration curves was purchased from 365 by Whole Foods Market (Austin, TX, USA). A certified reference material (CRM), ERM-BC211 rice flour, was obtained from the Joint Research Centre CRMs Catalog (Institute for Reference Materials and Measurements, Geel, Belgium). Commercial rice and rice-based foods were obtained from various sources in the U.S. market. Prior to use, all samples were milled into a powder (High Speed Multifunctional Disintegrator, Linyi Bright Star Food Machine Co., Linyi, China) and ground with a mortar and pestle. Calrose rice and commercial samples were kept at ambient storage conditions (20 °C) and the CRM was placed in frozen storage (−20 °C) while not in use.

### 2.2. ED-XRF Instrumentation

A Benchtop Epsilon 4 Energy Dispersive X-ray Spectrometer (Malvern PANalytical, Malvern, UK) was used to collect XRF spectra from all samples. The Epsilon 4 is capable of producing maximum tube voltage and current tube energy of 50 kV and 2 mA, respectively, operating at a maximum of 10 W. The X-ray tube comprises a silver (Ag) anode with a 50 µm beryllium window, and a high-resolution silicon drift detector with a resolution of 0.135 keV (at Mn Kα) enables a maximum count rate of 1,500,000 counts per second at 50% dead time [13]. Epsilon software (version 2.1.a, Malvern PANalytical) was utilized for initial spectral data collection.

### 2.3. ICP-MS Analysis

As and Pb content in the Calrose rice units, CRM, and commercial food samples used for model calibration and validation were quantified via ICP-MS at the UMass Amherst Mass Spectrometry Core Facility, RRID:SCR_019063, with support from the Massachusetts Life Science Center and Institute for Applied Life Sciences. The Association of Official Analytical Chemists Method for Heavy Metal Quantification in Foods by ICP-MS was used as a reference method for sample preparation and analysis [39].

A 0.5 g sample was weighed into a microwave digestion vessel with 4 mL of HNO_3_ concentration and 1 mL of 30% H_2_O_2_ (Sigma-Aldrich). A total of 0.1 mL of a 50 mg/L Lu solution in 5% nitric acid (Sigma-Aldrich) was spiked into each digestion vessel to assess potential analyte loss during digestion procedures. A CEM MARS5 microwave digestion system (Matthews, NC, USA) was employed for sample digestion with the following program: ramp to 145 °C, hold for 1 min; ramp to 50 °C, hold for 1 min; ramp to 145 °C, hold for 1 min; ramp to 170 °C, hold for 10 min; ramp to 190 °C, hold for 10 min. After digestion, samples were allowed to cool to room temperature, diluted fourfold with DI water, then diluted fourfold with a 1% nitric acid solution. All samples were analyzed in duplicate and sample blanks were prepared following the entire sample preparation method. Calibration standards for As and Pb (0–2.75 µg/L) were prepared through the dilution of a Perkin-Elmer multi-element certified reference standard in 1% nitric acid (Perkin-Elmer, Waltham, MA, USA). Samples were analyzed using a Perkin-Elmer NexION 350D ICP-MS utilizing ^75^As and ^208^Pb as reference isotopes for quantification. As and Pb concentrations were quantified via calibration curve slope interpolation. Recovery percentages of the certified reference standard solutions used to generate ICP-MS calibration curves are provided in the Supplementary Information (SI; Table S1). Method LOQ was calculated for both the As and Pb calibration curves through the following equation:

(1) $$\mathrm{LOQ}=10\times \;\frac{\sigma_{S}}{b}$$

where $\sigma_{S}$ is the standard deviation of the sample blanks and b is the slope of the calibration curve.

### 2.4. Sample Preparation and ED-XRF Analysis

Calibration standards were created through the spiking of milled and ground Calrose rice (average particle size ~300 µm) with varying concentrations and combinations of As and Pb at an elemental concentration range ~0–600 µg kg^−1^. The rice was accurately weighed into Falcon tubes (Fisher Scientific, Waltham, MA, USA), spiked with a diluted solution of DMA and/or lead (II) acetate trihydrate, and thoroughly mixed to ensure homogeneity. Discussion on the chemical forms of heavy metals, working concentration ranges, and the rice chosen for use as a calibration standard matrix are provided in SI Section S1.1 [4,5,6,7,11,13,40,41,42]. The final concentrations of As and Pb in calibrants were determined gravimetrically while also considering their background contamination levels as determined by ICP-MS (described in Section 2.3).

Pelletized samples were prepared from calibration standards as outlined in a previous study which thoroughly investigated the optimization of these sample preparation and ED-XRF analysis conditions [43]. Spiked standards were weighed into aluminum cups (4  ±  0.2 g, 31 mm length × 7.9 mm height, Premier Lab Supply, Port St. Lucie, FL, USA) and pelletized (pressure of 10 tons for 30 s each, 31 mm diameter × 5 mm thick). Three pellets were made from each calibration standard and analyzed in triplicate via ED-XRF with conditions displayed in Table 1. Analysis time per pellet was increased from 60 s to 120 s compared to the previous study [43] to enhance SNR around characteristic emission lines. Three pellets were analyzed in triplicate per standard, meaning that the total analysis time for a single standard was 1080 s. Resultant spectra were exported from the Epsilon 4 software for further data analysis.

### 2.5. ANN Model Development

A feed-forward ANN was used to build a nonlinear regression model for simultaneous prediction of As and Pb concentrations from ED-XRF spectra. To generate the calibration model, only those calibration standards developed in Section 2.4 were included as training data. Test data includes those discussed in later sections (Section 2.6 and Section 2.7). Calibration standards included those spiked with As, Pb, or both As and Pb. Spectra were background subtracted, meaning that total counts recorded at each energy channel were subtracted from background counts as determined via ED-XRF instrumentation. The nine background corrected spectra resulting from a single standard acted as one sample input but were left unaveraged when input into the model. Spectral regions around characteristic emission lines of As and Pb (As Kα/Pb Lα overlap region and Pb Lβ region) served as model inputs without explicitly associating each element with their characteristic emission line regions. Spectral regions were iteratively adjusted and the regions around each characteristic emission line yielding optimal calibration performance were selected for inclusion in the final calibration. Final spectral regions included for calibration model development include 10.35–10.70 keV and 12.40–12.85 keV (101 channels/spectra). The network architecture included two fully connected hidden layers with 256 and 128 neurons, respectively, employing rectified linear unit (ReLU) activation functions, dropout (*p* = 0.1), and full-batch gradient descent training to introduce nonlinearity and prevent overfitting [44]. The output layer had two neurons corresponding to As and Pb concentrations. Model training was conducted using the Adam optimizer (learning rate = 1 × 10^−5^) and the test root mean squared error (RMSE) as the loss function over 10,000 epochs. Best-model checkpointing was implemented as an early stopping strategy, as the model was evaluated on the test dataset every 500 epochs and the state yielding the lowest test RMSE was saved as the final model. Outliers resulting from standards with a residual z-score of greater than |2.5| were removed, in which the *z*-score was calculated by the following equation in Excel 2024 (Microsoft Office 2024):

(2) $$z_{r}=\;\frac{r}{\sigma_{r}}$$

where $z_{r}$ is the residual *z*-score, r is the calculated residual, and $\sigma_{r}$ is the standard deviation of the residuals. A *z*-score of |2.5| was the threshold value employed to ensure the removal of extreme outliers yet retain most data points which allowed the model to train on as many valid spectra as possible. Outlier exclusion included 4 standards (36 total spectra) and was performed prior to the development of the final calibration model. After outlier exclusion, the final calibration model was assessed and trained on 28 standards containing 252 total spectra. Detailed concentrations of As and Pb present in all standards used in calibration model development can be found in Table S2 (SI).

All ANN model development, training, and evaluation were performed using Python (version 3.12.12) in a Google Colaboratory environment. The neural network architecture was constructed and trained using the PyTorch deep learning library (version 2.10.0). Data preprocessing and manipulation were performed using NumPy (version 2.0.2) and pandas (version 2.2.2). Model evaluation metrics (R^2^, RMSE) were computed using scikit-learn (version 1.6.1).

### 2.6. Model Evaluation and Performance Metrics

Averages and standard deviations of peak areas around characteristic emission lines were determined to investigate signal variability. Calibration model metrics including regression line equations, linearity metrics, and the standard error of calibration (SEC) were calculated to assess calibration performance.

Experimental LODs and LOQs of the ANN calibration model were calculated under two analytical scenarios using methods and equations as outlined in the Eurachem Fitness for Purpose of Analytical Methods laboratory guide [45] as no true blanks were available during method development. The two scenarios included samples containing (1) detectable As and non-detectable Pb and (2) detectable As and Pb to investigate ANN predictive capabilities both in the presence and absence of significant spectral interference from Pb. In Scenario 1, 10 Calrose rice standards containing a spiked concentration of 128.97 µg kg^−1^ As were prepared and analyzed by ED-XRF. In Scenario 2, 10 Calrose rice standards containing spiked concentrations of 128.97 µg kg^−1^ As and 208.95 µg kg^−1^ spiked Pb were prepared and analyzed by ED-XRF. The respective spectra were input into the ANN model as test data to predict their elemental concentrations, and the results were utilized to calculate LOD/LOQ with the following equations:

(3) $$\mathrm{LOD}=3\cdot \;\frac{s_{o}}{\sqrt{n}}$$

(4) $$\mathrm{LOQ}=k_{Q\;}\cdot \;\frac{s_{o}}{\sqrt{n}}$$

where $s_{0}$ is the standard deviation of the predicted concentrations, *n* is the number of replicate observations averaged when reporting results, and $k_{Q\;}$ is a default factor of 10 set by the IUPAC which corresponds to an RSD of 10%, assuming the standard deviation in predictions is constant at low concentrations [45]. Bias in LOD/LOQ determinations was also calculated with the following equation:

(5) $$b=\;\bar{x}-x_{actual}$$

where b is predictive bias, $\bar{x}$ is the average of all predicted concentrations and $x_{actual}$ is the known concentration of As/Pb. In Scenario 1, only the LOD/LOQ of As were calculated and in Scenario 2, the LOD/LOQ of both As and Pb was calculated. A scenario in which samples contained non-detectable As and detectable Pb content was not included as such contamination is not commonly reported in rice and rice-based foods. These performance metrics reflect model-dependent limits as they are calculated through the standard deviation of results obtained through ANN modeling.

### 2.7. Validation

To test the trueness and real-world applicability of the method, a CRM (ERM-BC211) and eleven commercial rice and rice-based foods were obtained to act as validation data points. This CRM is a rice powder containing 260 ± 13 µg kg^−1^ total As (124 µg kg^−1^ inorganic As and 119 µg kg^−1^ organic As) as determined by high-performance liquid chromatography (HPLC)-ICP-MS at the Joint Research Centre Institute for Reference Materials and Measurements [46]. These samples were made into standards using methods described above (Section 2.4). Due to a lack of significant Pb content in each commercial sample, four samples were spiked with varying amounts of Pb (~75–400 µg kg^−1^) and made into standards. Three standards were made from four commercial samples at different Pb concentrations, generating twelve additional validation data points. Samples were pelletized and analyzed via ED-XRF, exported to the appropriate software(s), and processed according to the methods outlined above. Spectral data was input into the ANN model as test data and the reported predictions represent the average predictions for As and Pb from a single standard (*n* = 9).

Accuracy of model performance in validation samples was assessed by comparing the actual and predicted concentrations of As and Pb, in which a predefined error of ±20% between the actual and predicted values was deemed acceptable as per the FDA Office of Regulatory Affairs Methods, Method Verification and Validation guide [47]. In determining Pb error of unadulterated commercial samples, the actual error (µg kg^−1^) was calculated rather than percent (%) error due to low Pb content. Mean and median errors were calculated in each commercial food category to investigate and compare predictive capabilities at varying As and Pb concentrations.

## 3. Results and Discussion

### 3.1. ICP-MS Analysis of All Rice Samples Used Throughout the Study

Table 2 shows the ICP-MS results of As and Pb determinations made in the unadulterated Calrose rice units utilized in the development of calibration standards. The quantifications of total As were much higher those that of Pb and fall around the average values previously reported for the total As contamination in rice grown in various regions across the United States [40]. Total As determinations in these Calrose rice units, specifically Unit 2 with an average determination of 87.82 µg kg^−1^ As, enabled this method to be employed for As and Pb detection in line with current regulatory guidelines, as this value falls under the maximum allowable limits in most current regulatory literature [4,5,6,7]. When spiking As and Pb into all calibration standards, these determinations were considered and included in the final known concentrations.

Table 2 also displays the ICP-MS determinations in the CRM and commercial rice/rice-based foods used for validation purposes. The CRM was analyzed to quantify its unknown Pb content, however its average determination of As (263.63 µg kg^−1^) compared to the certified reference value (260 µg kg^−1^ total As) indicates the accuracy of the method. A wide range of rice-based products intended for the general population or targeted for consumption by infants and small children were obtained to determine the applicability of the method across unique rice-based matrices. These products contained a wide range of As content (56.84–296.72 µg kg^−1^) which was ideal as it allowed for the ANN model to demonstrate its predictive capabilities across a large concentration range. Ten of eleven commercial samples contained Pb content lower than the LOQ of the method, so as to demonstrate model predictive ability across a wider concentration range of Pb, four commercial samples of differing matrices were chosen to be spiked with varying amounts of Pb which covered a similar concentration range to that of As.

### 3.2. ED-XRF Spectra of Calibration Standards

**Figure 1:**
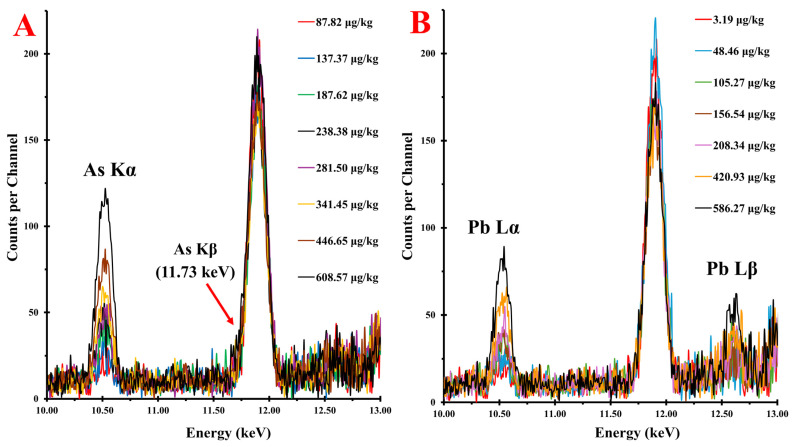
Averaged and background subtracted energy-dispersive X-ray fluorescence (ED-XRF) spectra around characteristic emission lines of calibration standards containing: (**A**) spiked arsenic (As); (**B**) spiked lead (Pb).

Calibration standards spiked with only As, only Pb, or a combination of As and Pb were analyzed by ED-XRF. Figure 1 displays the average spectra of select calibrants containing only spiked As (Figure 1A) or spiked Pb (Figure 1B) around their characteristic emission lines. Figure 1A demonstrates the sensitivity of the method in the detection of As, as the lowest concentration standard yielded a weak yet identifiable peak at its principal emission line (As Kα = 10.52 keV [22]) which is clearly shown in Figure S1 (SI). The spectra of As-containing calibration standards showed significant peak overlap from 87.82 to 341.45 µg kg^−1^ with clear peak separation in samples ranging from 446.65 to 608.57 µg kg^−1^ As. A discussion on As Kβ emissions and their inability to be employed for As quantification in this study is provided in the SI (Section S2.1 [11,22,48,49,50]).

Figure 1B shows the ED-XRF spectra of calibration standards spiked with Pb. Around its principal emission line (Pb Lα = 10.50 [22]), the spectra showed clear peak separation from 586.27 to 420.93 µg kg^−1^ Pb; however, there was a significant overlap in samples containing 208.34 to 3.19 µg kg^−1^ Pb. The identifiable peaks in samples containing the lowest Pb content were likely due to spectral emissions from natural As contamination present in the Calrose rice units, as the spectral overlap between Pb and As is observed at this energy value. An additional peak dependent on the Pb content was observed around 12.6 keV which is associated with the Pb Lβ line. While clear peaks were observed, they showed weaker intensity compared to their Lα counterparts which can be explained by transition probabilities, as Lα emission events are about 1.61 times more likely to occur than an Lβ event [11]. Though these emissions were relatively weak, which may result in worsened ability for regression models to accurately predict Pb content from its Lβ line, they were employed for Pb quantification in this study due to their lack of spectral overlap with other elements.

Comparing Figure 1A,B, it was observed that for the same concentration of spiked heavy metal, the standards containing As resulted in more intense spectra at the same emission energy (10.5 keV). This is due to the fluorescence yield (ω), or the probability that an X-ray photon of a given shell will be ejected upon sufficient excitation [11], in K- and L-shells. Though ω is positively correlated with atomic number, fluorescence yield values for K-shell emission are much greater than those of L-shell emission. The fluorescence yield of an As K-shell emission is 0.549, whereas ω = 0.386 for Pb L-shell emission, yielding more intense emissions for As around its Kα line when compared to the same concentration of Pb around its Lα line. The ω of Pb K-shell emission is 0.953 and would result in much more intense spectra compared to the K-shell emissions of As, however the energy required to eject an electron from this shell (Pb Kα = 73.89 keV [22]) is much too high for the utilized ED-XRF spectrometer in this study to achieve [13].

### 3.3. ANN Calibration Performance

#### 3.3.1. Calibration Metrics

**Figure 2:**
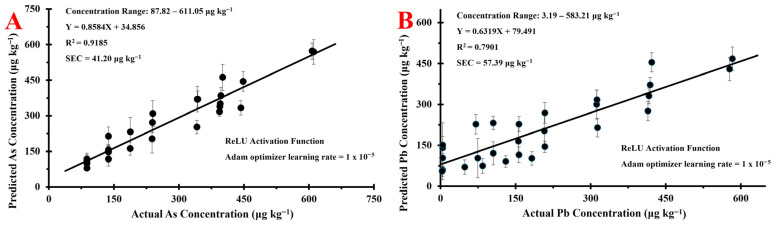
Artificial neural network (ANN) calibration curves comparing predicted and actual concentrations of: (**A**) As; (**B**) Pb.

Figure 2 shows calibration regression plots for As (A) and Pb (B) which were generated utilizing an ANN with the ReLU activation function. Spectral regions included during final calibration model training include 10.35–10.70 keV and 12.40–12.85 keV (101 channels/spectra). Data points on the regression curves represent the average of 9 predictions as spectra were left unaveraged before being input into the ANN, with error bars included to represent the standard deviation within a calibration standard. Calibration metrics for As were great, yielding an R^2^ = 0.92 and SEC = 41.20 µg kg^−1^. This correlates to an SEC = 6.74% relative to the working concentration range, further demonstrating strong agreement between observed and actual As concentrations across the entire concentration range. The strong performance of the As calibration model was likely due to the ANN’s precise ability to attribute spectral regions to the correct element and the inherent sensitivity of As detection around its Kα line. Though analyte-specific regions were not explicitly attributed to each element, the ANN was able to relate spectral data to As across the entire concentration range. This indicated a great ability of the ANN to correspond As concentration to emissions around its Kα line without strong correlation with the emissions attributed to Pb around its Lβ line. A second reason for the strong capabilities of the ANN to relate spectral data to As content is the inherent sensitivity of As detection around its Kα line, as fluorescence yields are 1.42 times greater than Pb emissions at the same energy line (Pb Lα) [11]. Instrumental detection of As was found to be very sensitive through the developed method, allowing the ANN to recognize emissions attributed to As even at the lowest concentrations investigated in this study.

Metrics were worsened for Pb (Figure 2B) which resulted in poor goodness-of-fit (R^2^ = 0.79) and error metrics (SEC = 57.39 µg kg^−1^, relative SEC = 9.84%). This calibration also shows clear bias and the inability to accurately relate spectral data to Pb content across the entire concentration range. At low concentrations, the calibration overestimates Pb with error reaching as high as +156.81 µg kg^−1^ between predicted and actual concentrations. Conversely, Pb content is underestimated as the concentration increases with error reaching as high as −147.99 µg kg^−1^. This bias is further highlighted in its error plot (SI; Figure S2) showing a highly linear and negative correlation (R = 0.75, slope = −0.3681) between error values and Pb concentration, indicating significant proportional bias and slope compression across the entire concentration range. The clear bias and slope compression will likely affect its ability to accurately predict Pb content in unseen data, affecting its overall performance in the assessment of its validation.

Performance in the Pb calibration was weaker compared to the As counterpart due to how the spectral data was inputted into the model for training and the inherent sensitivities of Pb emission lines. In the development of the calibration sets, the 9 ED-XRF spectra resulting from a single standard were not averaged before being input into the ANN model in order to allow the model to train on variability within samples as well as between samples. Compared to traditional chemometric regression models, ANNs require much more data to train a robust calibration set due to the high number of training parameters, including weights and biases, which are used within hidden layers to generate nonlinear relationships between the input and output values [38]. In allowing the ANN to train on more data through leaving spectra unaveraged, this consequentially reduced the SNR associated with each data point, leading to a worsened ability for the ANN to properly model relationships between spectral data and elemental content across the entire concentration range. These factors, in addition to the inherently weak sensitivity in Pb detection around its Lα and Lβ emission lines (discussed in Section 3.2), reduced the ability of the ANN to learn minute spectral features which differentiated samples especially at low concentrations, resulting in poor calibration performance.

#### 3.3.2. Experimental Determinations of LOD and LOQ

Experimental LOD and LOQ determinations calculated for the ANN calibration model are shown in Table 3. Details on the exact predicted concentrations of As and Pb in all 10 standards used in each analytical scenario are found in Table S3 (SI). Calculated metrics for As in the presence of non-detectable Pb (Scenario 1) are great, as the low standard deviation of predictions (9.39 µg kg^−1^) allowed for an As LOD and LOQ of 16.26 and 54.20 µg kg^−1^, respectively. An LOD of 16.26 µg kg^−1^ outperforms those obtained by Tatsumi et al. (2016) and Tsuyama et al. (2025) which utilized high-powered wavelength dispersive XRF and costly total reflection XRF spectrometers to detect As in rice and rice-based foods, respectively [16,51]. Additionally, these metrics slightly outperform those obtained by Chen et al. (2025) which obtained an LOD of 20 µg kg^−1^ in the analysis of As in various grain types utilizing a high-powered monochromatic ED-XRF spectrometer [31]. Compared to the previous studies discussed, the present work utilizes a low-cost and low-energy benchtop ED-XRF spectrometer while achieving comparable and/or stronger detection limits, highlighting both the sensitivty of the developed method and precision of the ANN modeling. However, the average prediction of 113.69 µg kg^−1^ As when compared to the known value of 128.97 µg kg^−1^ As (bias = −15.38 µg kg^−1^ or −11.85%) suggests a slight bias of the model to under-report As at this concentration in the presence of non-detectable Pb. The LOD/LOQ for As quantification meets current regulatory guidelines [4,5,6,7], however a slight bias of the model to underestimate As content in the presence of low Pb content may impact performance in validation agreement.

Table 3 also displays the LOD/LOQ determinations for As and Pb when both elements were present in detectable concentrations (Scenario 2) in a rice-based matrix. Detection and quantification limits calculated for As are similar to those obtained in Scenario 1, further indicating a strong ability for the ANN model to correctly relate spectral emissions to their respective elements (discussed in Section 3.3.1). However, an average As prediction of 164.90 µg kg^−1^ (bias = +35.92 µg kg^−1^ or +27.86%) across all 10 samples indicates a tendency of the ANN model to overestimate elemental content at this As-Pb concentration as the true value was 128.97 µg kg^−1^. While the low standard deviation in predictions enabled strong LOD/LOQ metrics for As in the presence of significant Pb interference, these results are hindered by a significant bias to overestimate its elemental content in this analytical scenario which suggests concentration-dependent accuracy limitations despite the precise nature of the ANN model.

Detection and quantification limits were also calculated for Pb in analytical scenario 2 (Table 3). Though these metrics are worsened compared to those obtained for As, the ANN still demonstrated high precision across all 10 determinations, resulting in an LOD = 25.87 µg kg^−1^ and LOQ = 86.23 µg kg^−1^, respectively. However, these results indicate a significant bias to underestimate Pb content at this As-Pb concentration range (bias = −74.08 µg kg^−1^ or −35.46%). The bias in results significantly hinders the quantitative reliability for Pb determinations and was expected due to the poor calibration for Pb (discussed in Section 3.3.1). Similar to the metrics associated with As in this analytical scenario, the high precision of the ANN model yielded strong LOD/LOQ metrics, however significant biases in the observed predictions suggest accuracy limitations which reduce its reliablility in trace determinations of Pb content at this As/Pb concentration range.

### 3.4. Validation Performance

Table S4 (SI) displays the detailed predictions of As and Pb made by the ANN model in all validation samples compared to their known values while Table 4 displays the summarized results. The agreement in the CRM’s As determination was valid with an error of −19.43%, indicating its ability to accurately quantify trace As in a rice-based matrix. While this prediction (209.50 µg kg^−1^ As) does not fall within the CRM’s uncertainty threshold (260 ± 13 µg kg^−1^ [46]), the result was still considered valid as it falls within the ±20% threshold used to assess agreement in all validation results [47].

For commercial rice/rice-based food samples, where the Pb concentration was non-detectable and As was found at detectable concentrations in all samples, the ANN showed good agreement between actual and predicted concentrations (absolute average error = 20.69%). Samples containing higher As concentrations (154.10–296.72 µg kg^−1^, *n* = 5) all yielded negative errors greater than ±20%, indicating disagreement and a slight bias to underestimate As content in commercial foods containing higher As and non-detectable Pb. Though As was underestimated in high As-containing samples, As determinations in samples containing both low As and Pb were very agreeable with an absolute average error of only 9.07 µg kg^−1^ in the 6 samples containing the lowest As content. Error in the Rice Wafer sample was +30.33% which indicated disagreement between predicted and actual values; however, an error of only +17.24 µg kg^−1^ was strong considering the low actual As content in this sample.

For samples containing spiked Pb (75.57–416.67 µg kg^−1^ Pb), the ANN model demonstrated a very strong ability to accurately predict As with an absolute average error of 14.11% and average error of −1.15% between predicted and actual concentrations. 8 of the 12 Pb-spiked samples yielded less than ±20% error in As determinations with no samples showing higher than ±30% error. The great agreement in As predictions within samples containing spiked Pb further highlights the ANN’s ability to correctly attribute spectral emissions to the correct analyte when both elements are present at detectable levels.

Table 4 and Table S4 (SI) also show the predictions of Pb across all samples employed for method validation. The actual concentrations of Pb in the CRM (5.28 µg kg^−1^ Pb) and 10 of 11 commercial samples were below the calculated LOQ (Table 3), indicating that these results are not quantitatively meaningful in regard to the developed method. However, an observed prediction bias in these samples further highlights the inability of the ANN to accurately quantify trace Pb in a rice-based matrix. An average error of +81.11 µg kg^−1^ Pb suggests that the model consistently overestimates trace Pb content, aligning with the observed trends in the Pb calibration set (Figure 2B). Further, in Pb-spiked samples containing Pb content near or above the calculated LOQ (Table 3), the ANN showed a poor ability to accurately quantify Pb with a median absolute error of 35.00%. One sample (Rice Cereal #2–Spiked with ~125 µg kg^−1^ Pb) achieved an error less than ±20%, however all other samples containing Pb content above the Scenario 2 LOQ (Table 3) resulted in disagreement between their predicted and known Pb content. Additionally, a negative proportional bias is observed in the results, as the Pb error plot in the Pb-spiked commercial foods, shown in SI Figure S3, shows a negative linear correlation between actual Pb content and prediction error (R = 0.9343, slope = −0.8345). This bias indicates that Pb content will be overestimated at low concentrations and underestimated at high concentrations, following the same trends found in both the calibration plot and results obtained during experimental determinations of LOD/LOQ (Section 3.3.1 and Section 3.3.2, respectively). Overall, the developed ED-XRF-ANN method demonstrates a great ability to accurately quantify As in rice-based foods both in the absence and presence of significant spectral interference from Pb; however, accurate quantifications of Pb were not observed across the entire concentration range.

### 3.5. Strengths, Limitations, and Future Work

The present work indicates that ANNs are an effective tool in enabling accurate determinations of trace As in the absence and presence of significant spectral interference from Pb. Conventional approaches, including fundamental parameters and univariate or multivariate linear regression tools, have also been explored for enabling accurate quantifications of As via XRF in the presence of spectral interference in various matrices [28,29,30,31]. While these models have been shown to be effective under high SNR conditions in which the relationships between As and Pb concentrations and XRF signal are linear, their performance may be weakened in low SNR environments when these rigid relationships are less predictable, dominated by noise and matrix-dependent background signals. This highlights the advantages of ANN modeling, as it was able to effectively capture these complex and potentially non-linear relationships in low SNR environments. However, an advantage of one of the aforementioned studies employing fundamental parameters for spectral deconvolution [31] is its ability to accurately quantify both As and Pb in grain-based matrices. Due to poor ANN modeling of Pb which yielded significant biases and an inability to accurately relate spectral features to analyte concentration across the entire concentration range investigated, additional studies can explore the use of traditional or innovative chemometric data modeling approaches to improve simultaneous quantification of As and Pb, enhancing method robustness and quantitative reliability for multiple heavy metals in a rice-based matrix.

A second limitation of the method presented in this study is the inability to differentiate between chemical species of As. Most regulations pertaining to actionable limits on As in these foods specify limitations on iAs, as these species are known to be more toxic than their organic counterparts [4,5,6,7]. Therefore, it is intended that this method may be applied for preliminary screening of total As in these foods to aid in the assessment of samples which may require additional speciation analysis to confirm that iAs levels comply with current regulatory guidelines. Future studies could explore additional sample preparation techniques to enable chemical speciation of As; however, the present work remains a rapid, cost-effective, and accurate tool for total As screening in rice-based foods.

## 4. Conclusions

This study demonstrates that coupling ED-XRF with ANN modeling provides a viable strategy for correcting spectral overlap between the As Kα and Pb Lα emission lines, enabling accurate quantifications of As in rice-based foods. The ANN-based calibration exhibited great performance for As across the full concentration range evaluated, enabling accurate predictions even at trace levels and in the presence of detectable Pb. Validation using diverse rice-based food products confirmed that the ANN effectively mitigated As–Pb spectral interference, resulting in reliable and robust As determinations. Overall, ANN modeling demonstrated a poor ability to relate spectral data to Pb content, resulting in poor calibration metrics and inaccuracies across all validation studies. This limitation is attributed to the inherently weak Pb L-shell emissions and their low SNRs in rice-based matrices. Overall, this work demonstrates that ED-XRF combined with ANN modeling offers a rapid, minimally destructive, and cost-effective approach for trace As screening in rice-based foods, even when Pb is present and spectrally overlapping. However, achieving robust quantitative Pb predictions in these matrices remains challenging. Future studies will be needed to further improve Pb quantification in the presence of As in agricultural and food products.

## Figures and Tables

**Table 1:** ED-XRF analysis conditions.

Parameter	Parameter Setting
X-ray source voltage	50 kV
Tube current	0.2 mA
Filter	Ag
Analysis time	120 s
Medium	Air
Detector mode	Normal

**Table 2:** As and Pb determinations in all rice samples used throughout this study as determined by inductively coupled plasma mass spectrometry (ICP-MS).

Sample Name	^75^Arsenic (μg kg^−1^)		^208^Lead (μg kg^−1^)	
	Average Determination	Standard Deviation	Average Determination	Standard Deviation
Calrose Rice Unit 1	137.37	2.03	4.26 *^a^*	0.30
Calrose Rice Unit 2	87.82	2.70	3.19 *^a^*	0.37
CRM (ERM-BC211)	263.63	1.27	5.28 *^a^*	0.06
Brown Rice Cereal #1	296.72	9.13	0.00 *^a^*	0.44
Rice Cereal #1	248.03	0.75	0.00 *^a^*	0.20
White Rice	225.85	12.22	0.00 *^a^*	2.10
Brown Rice	183.10	0.36	0.18 *^a^*	0.35
Rice Cereal #2	154.10	9.30	0.00 *^a^*	1.12
Rice Rusks #1	127.69	6.80	1.23 *^a^*	4.00
Rice Cereal #3	125.97	0.18	0.00 *^a^*	0.25
Rice Puffs #1	101.71	2.33	0.00 *^a^*	2.61
Rice Rusks #2	99.06	1.87	0.00 *^a^*	1.51
Rice Puffs #2	73.10	2.67	0.00 *^a^*	0.67
Rice Wafer	56.84	2.60	28.71	27.20

*^a^* Determination less than the Pb LOQ (LOQ = 6.59 μg kg^−1^ Pb).

**Table 3:** Calculated limits of detection (LODs), quantification (LOQs), and bias of the ANN calibration models in two analytical scenarios (all units in µg kg^−1^).

Scenario	Element	Actual Concentration	Average Predicted Concentration (*n* = 10)	Bias in Predictions	Standard Deviation of Predictions	Calculated LOD	Calculated LOQ
Detectable As and non-detectable Pb (1)	As	128.97	113.69	−15.38	9.39	16.26	54.20
	Pb	–	–	–	–	–	–
Detectable As and Pb (2)	As	128.97	164.90	+35.92	9.21	15.95	53.17
	Pb	208.95	134.87	−74.08	14.94	25.87	86.23

**Table 4:** Summarized prediction accuracy in all validation samples employing the ANN for As and Pb quantifications.

Sample Category	Absolute As Error		Absolute Pb Error	
	Mean	Median	Mean	Median
CRM (ERM-BC211) (*n* = 1) ^a^	19.43%		151.53 µg kg^−^1 ^b^	
Commercial rice and rice-based foods (*n* = 11)	20.69%	25.70%	81.11 µg kg^−1 b^	84.02 µg kg^−1 b^
Commercial foods spiked with Pb (*n* = 12)	14.11%	11.17%	44.24%	35.00%

^a^ Reported error does not contain a mean or median value as *n* = 1. ^b^ Actual Pb content is lower than the LOQ determined via ICP-MS; therefore, the error is not quantitatively meaningful under experimental conditions.

## Data Availability

The raw data supporting the conclusions of this article will be made available by the authors on request.

## Supplementary Materials

The following supporting information can be downloaded at https://www.mdpi.com/article/10.3390/foods15071130/s1, Additional Materials and Methods: S1.1. Sample preparation and ED-XRF analysis; Additional Results and Discussion: S2.1. ED-XRF spectra of calibration standards; Table S1: Recovery of calibration standards utilized in inductively coupled plasma mass spectrometry (ICP-MS) analysis; Table S2. Concentrations of As and Pb in calibrant standards utilized in calibration model development. Figure S1: Averaged and background subtracted ED-XRF spectra of the lowest and highest As-containing calibration standards around the characteristic emission lines of As; Figure S2: Error plot generated from the Pb ANN calibration curve; Table S3: Detailed predictions of As and Pb in 10 standards at varying Pb levels used in experimental determinations of LOD and LOQ (all units in µg kg^−1^); Table S4: Prediction accuracy in all validation samples employing an ANN for As and Pb quantification. Figure S3: Error plot generated from Pb determinations in Pb-spiked commercial foods.

## Abbreviations

The following abbreviations are used in this manuscript:

ANN	artificial neural network
As	arsenic
CRM	certified reference material
DMA	dimethylarsinic acid
ED-XRF	energy dispersive X-ray fluorescence spectrometry
ICP-MS	inductively coupled plasma mass spectrometry
LOD	limit of detection
LOQ	limit of quantification
Pb	lead
R^2^	coefficient of determination
ReLU	rectified linear unit
RMSE	root mean squared error
SEC	standard error of calibration
SNR	signal-to-noise ratio
