# Supplementary material for: Rapid and Accurate ED-XRF Quantification of Trace Arsenic in Rice-Based Foods Employing ANNs to Resolve Lead Spectral Interference
Source: Foods. 2026 Mar 25;15(7):1130. doi: 10.3390/foods15071130 (PMC13072912; doi:10.3390/foods15071130)
Supplement: Supplementary file 1 [file foods-15-01130-s001.zip › foods-4167288-supplementary.pdf]

S1. MATERIALS AND METHODS

S1.1. Sample preparation and ED-XRF analysis

While DMA and lead (II) trihydrate were the specific chemical forms of As and Pb utilized as spiking agents, respectively, literature supports that the oxidation state or chemical structure in which these elements are present do not significantly affect the resultant ED-XRF spectra, especially when considering the analysis of emission lines resulting from inner shell (K and L shells) events and the resolution of the utilized spectrometer [11, 13]. The upper concentration range limit of 600 μg kg^{-1} was determined based on current regulatory guidelines outlining maximum permissible limits for As and Pb in rice cereals [4-7], as quantifications above this limit would fall outside the scope of regulatory relevancy. Calrose rice was chosen as the rice type used in the development of calibration standards as rice grown in California has been shown to contain lower amounts of As than other regions of the United States [40-42]. An enriched form of this medium-grain white rice variety was obtained to mimic the nutrient profile of many enriched rice-based foods intended for infants and small children.

S2. RESULTS AND DISCUSSION

S2.1. ED-XRF spectra of calibration standards

While yielding intense peaks around its principal emission line, As Kβ emissions around 11.73 keV were not clearly defined across the entire concentration range. A significant peak at 11.9 keV was present in all samples, likely due to trace contamination of Calrose rice units with Br (Br Kα = 11.90 keV [22]) which has been commonly reported [48-50]. Though a slight peak tail is observed around the As Kβ line in high As-spiked calibration standards (Fig. S1), it was found that these tails indicating the presence of As were not significant enough to extract meaningful

information across the entire concentration range investigated. Emissions in a given sample around the Kβ line of As are less intense compared to its Kα line due to inherent transition probabilities. Kα emission lines result from an event in which an electron from the K-shell is ejected and the vacancy is filled by L-shell (next outer-most shell) electron, whereas Kβ lines results from an M-shell (second outer-most shell) electron filling the K-shell vacancy [11]. The probability of the L to K-shell transition is more probabilistic than the M to K-shell due to proximity and electron shell energy differences [11]. Therefore, due to the significant overlap with an observed Br peak as well as reduced intensity when compared to its principal emission line, the As Kβ line could not be employed for As quantification in this study.

Table S1. Recovery of calibration standards utilized in inductively coupled plasma mass spectrometry (ICP-MS) analysis

Nominal Standard Concentration (µg kg^{-1})	Calculated Standard Concentration (µg kg^{-1})		Recovery	
	As	Pb	As	Pb
0.000	0.000	0.006	-	+0.006 µg kg^{-1}
0.050	0.050	0.050	100.00 %	100.00 %
0.100	0.094	0.096	94.06 %	95.71 %
0.250	0.252	0.245	100.85 %	98.01 %
0.750	0.751	0.744	100.20 %	99.22 %
1.750	1.735	1.726	99.14 %	98.62 %
2.750	2.760	2.760	100.37 %	100.37 %

Table S2. Concentrations of As and Pb in calibrant standards utilized in calibration model development

Standard Number	As Concentration ($\mu g\ kg^{-1}$)	Pb Concentration ($\mu g\ kg^{-1}$)
1	87.82	3.19
2	87.82	48.46
3	87.82	84.05
4	87.82	130.31
5	87.82	182.37
6	137.37	4.26
7	137.37	74.39
8	137.37	105.27
9	137.37	156.54
10	137.37	208.34
11	137.37	313.54
12 [a]	13.37	420.93
13	187.62	4.26
14	187.88	155.78
15 [a]	188.52	413.47
16	238.38	4.26
17	238.89	207.31
18	239.93	414.52
19	341.45	4.26
20	342.5	311.95
21	343.56	416.63
22	393.94	105.23
23	394.73	156.64
24	395.51	208.32
25	397.1	312.47
26	401.14	577.6
27	442.95	70.47
28 [a]	446.65	4.26
29	448.25	418.77
30	607.34	422.01
31[a]	608.57	4.26
32	611.05	583.21

[a]Standard identified as an outlier during ANN calibration model development ($z_r > |2.5|$) and was removed before final calibration model training

Figure S1. Averaged and background subtracted ED-XRF spectra of the lowest and highest As-containing calibration standards around the characteristic emission lines of As

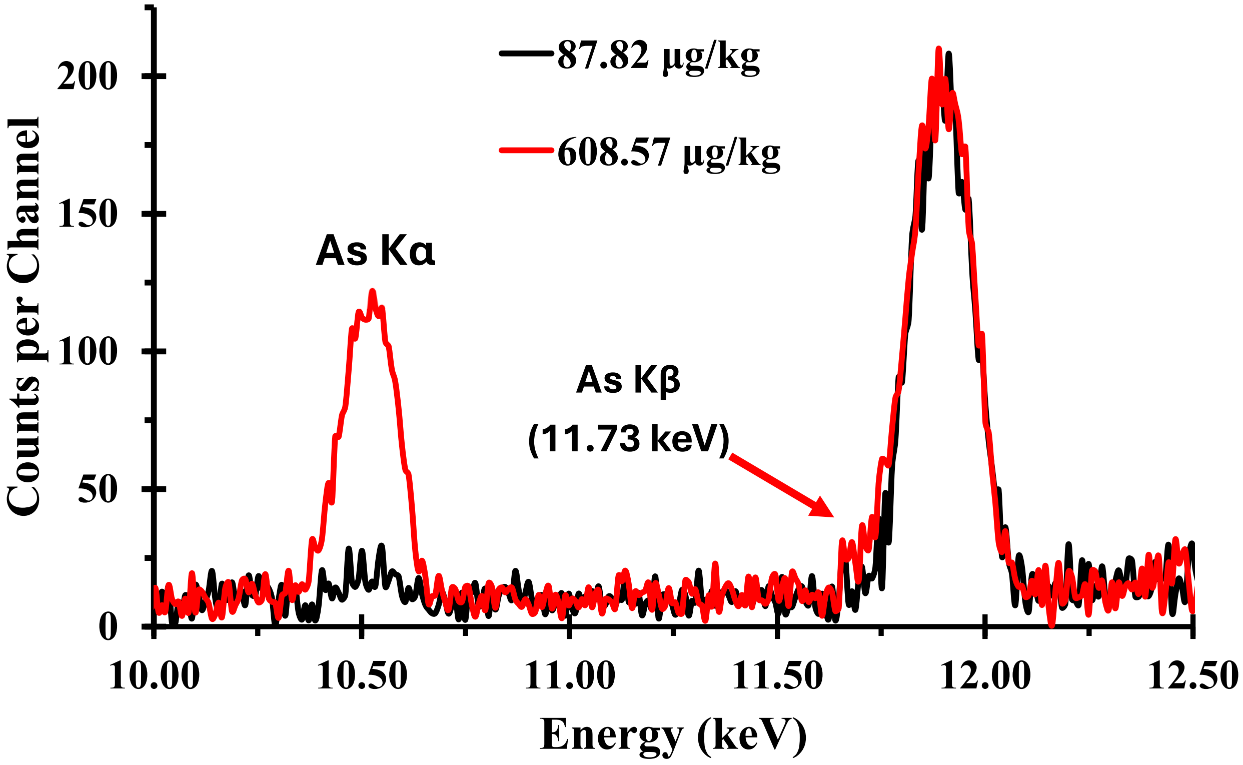

Figure S2. Error plot generated from the Pb ANN calibration curve

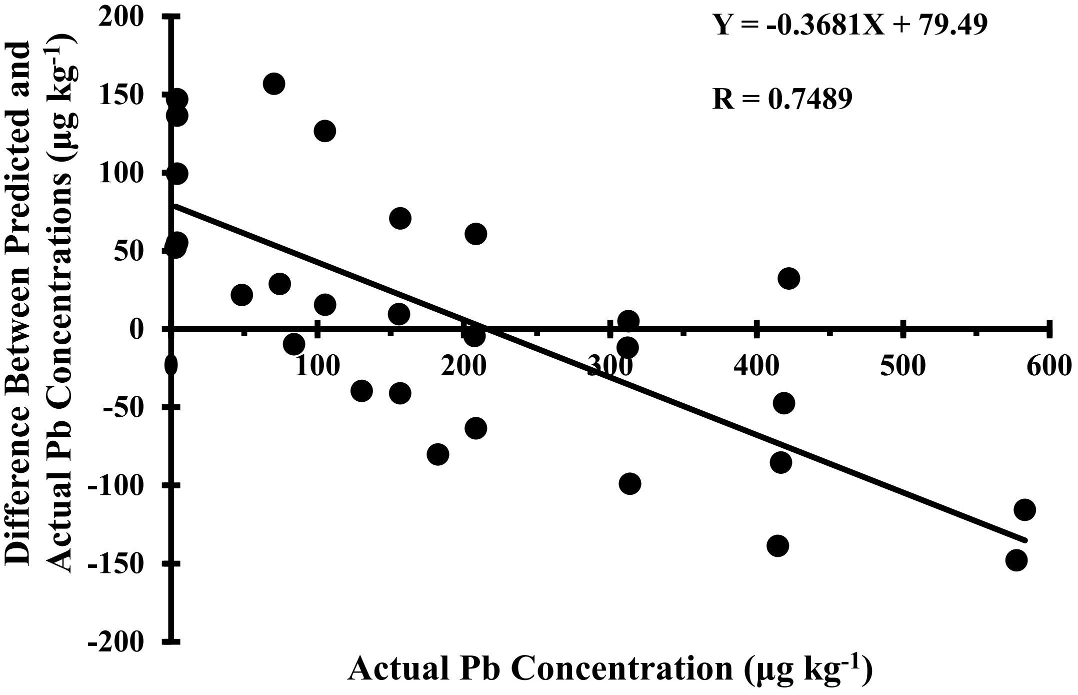

Table S3. Detailed predictions of As and Pb in 10 standards at varying Pb levels used in experimental determinations of LOD and LOQ (all units in µg kg^{-1})

Scenario	Element	Actual Elemental Concentrations	Predicted Elemental Concentrations	Average Predicted Concentration
Detectable As and non-detectable Pb (1)	As	128.97	118.12	113.69
			106.38	
			131.40	
			102.25	
			126.64	
			110.79	
			113.73	
			112.73	
			104.63	
			110.10	
	Pb	–	–	–
Detectable As and Pb (2)	As	128.97	169.96	164.90
			161.99	
			164.84	
			162.56	
			171.58	
			160.95	
			165.83	
			151.08	
			155.61	
			184.54	
	Pb	208.95	158.87	134.87
			118.81	
			124.19	
			115.86	
			136.88	
			136.99	
			136.11	
			131.61	
			129.10	
			160.25	

Table S4. Prediction accuracy in all validation samples employing an ANN for As and Pb quantification

Sample Type	Sample Name	As Determinations (µg kg^{-1})		Pb Determinations (µg kg^{-1})		Error		Error Metrics Grouped by Sample Type			
								As		Pb	
		Actual	Predicted	Actual	Predicted	As (%)	Pb	Absolute Average Error	Absolute Median Error	Absolute Average Error	Absolute Median Error
Reference Material	CRM (ERM-BC211)	260.00	209.50	5.28	156.81	-19.43	+151.53 µg kg^{-1} [a]	-	-	-	-
	Brown Rice Cereal #1	296.72	189.97	0.00	125.83	-35.98	+125.83 µg kg^{-1} [a]				
	Rice Cereal #1	248.03	151.82	0.00	90.63	-38.79	+90.63 µg kg^{-1} [a]				
	White Rice	225.85	155.84	0.00	102.02	-31.00	+102.02 µg kg^{-1} [a]				
	Brown Rice	183.10	136.05	0.18	90.01	-25.70	+89.83 µg kg^{-1} [a]				
	Rice Cereal #2	154.10	111.27	0.00	84.02	-27.79	+84.02 µg kg^{-1} [a]				
Commercial rice and rice-based foods	Rice Rusks #1	127.69	120.47	1.23	85.79	-5.65	+84.56 µg kg^{-1} [a]	20.69 %	25.70 %	81.11 µg kg^{-1}	84.02 µg kg^{-1}
	Rice Cereal #3	125.97	115.09	0.00	76.92	-8.64	+76.92 µg kg^{-1} [a]				
	Rice Puffs #1	101.71	100.71	0.00	72.62	-0.98	+72.62 µg kg^{-1} [a]				
	Rice Rusks #2	99.06	93.47	0.00	69.01	-5.65	+69.01 µg kg^{-1} [a]				
	Rice Puffs #2	73.10	85.59	0.00	68.98	+17.08	+68.98 µg kg^{-1} [a]				
	Rice Wafer	56.84	74.08	28.71	56.55	+30.33	+27.85 µg kg^{-1}				
	Brown Rice Cereal #1 – ~225 µg kg^{-1} Spiked Pb	296.72	208.56	230.18	156.33	-29.71	-32.08 %				
	Brown Rice Cereal #1 - ~150 µg kg^{-1} Spiked Pb	296.72	270.55	152.28	207.34	-8.82	+36.15 %				
	Brown Rice Cereal #1 - ~75 µg kg^{-1} Spiked Pb	296.72	234.72	75.57	170.13	-20.90	+125.14 % [b]				
	White Rice - Spiked with ~400 µg kg^{-1} Pb	225.85	262.12	416.67	209.07	+16.06	-49.82 %				
	White Rice - Spiked with ~300 µg kg^{-1} Pb	225.85	244.76	309.28	204.59	+8.37	-33.85 %				
	White Rice - Spiked with ~200 µg kg^{-1} Pb	225.85	216.22	204.08	157.56	-4.26	-22.79 %				
Commercial foods spiked with Pb	Brown Rice - Spiked with ~350 µg kg^{-1} Pb	183.10	237.35	362.87	186.90	+29.63	-48.49 %	14.11 %	11.17 %	44.24 %	35.00 %
	Brown Rice - Spiked with ~250 µg kg^{-1} Pb	183.10	186.28	256.59	173.39	+1.74	-32.42 %				
	Brown Rice - Spiked with ~175 µg kg^{-1} Pb	183.10	173.06	178.30	128.00	-5.48	-28.21 %				
	Rice Cereal #2 – Spiked with ~325 µg kg^{-1} Pb	154.10	187.95	335.92	148.72	+21.97	-55.73 %				
	Rice Cereal #2 – Spiked with ~200 µg kg^{-1} Pb	154.10	139.43	204.08	108.08	-9.52	-47.04 %				
	Rice Cereal #2 – Spiked with ~125 µg kg^{-1} Pb	154.10	134.35	126.58	102.30	-12.82	-19.18 %				

[a]Actual Pb content is lower than the ICP-MS LOQ

[b]Actual Pb content lower than the calculated LOQ (Table 3, Scenario 2)

Figure S3. Error plot generated from Pb determinations in Pb-spiked commercial foods

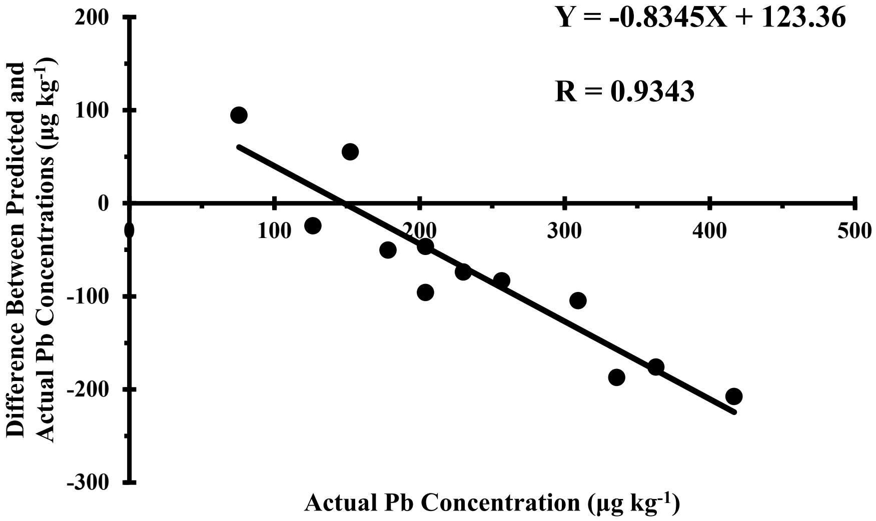

S3. REFERENCES

4. Center for Food Safety and Applied Nutrition. *Guidance for Industry: Action Level for Inorganic Arsenic in Rice Cereals for Infants*; Docket Number FDA-2016-D-1099; Food and Drug Administration: College Park, MD, USA, 2020.
5. European Commission. *Commission Regulation (EU) 2023/915*; Document 32023R0915; Official Journal of the European Union: Luxembourg, 2023.
6. *GB 2762-2025*; National Food Safety Standard Maximum Levels of Contaminants in Foods. Chinese National Health Commission: Beijing, China, 2025.
7. Food Safety and Standards Authority of India. *Food Safety and Standards (Contaminants, Toxins and Residues) Regulations, 2011*; Document Version-VIII; Food Safety and Standards Authority of India: New Delhi, India, 2025.
11. Haschke, M.; Flock, J.; Haller, M. *X-Ray Fluorescence Spectroscopy for Laboratory Applications*; Wiley, Hoboken, NJ, USA, 2021; pp. 1–464. https://doi.org/10.1002/9783527816637.
13. Malvern PANaytical—Epsilon 4. Available online: https://www.malvernpanalytical.com/en/products/product-range/epsilon-range/epsilon-4 (accessed on 3 September 2025).
22. Thompson, A.C.; Attwood, D.T.; Gullikson, E.M.; Howells, M.R.; Kim, K.-J.; Kirz, J.; Kortright, J.; Lindau, I.; Pianetta, P.; Robinson, A.; et al. *X-Ray Data Booklet*; Lawrence Berkeley National Laboratory: Berkeley, CA, USA, 2009.
40. Tatahmentan, M.; Nyachoti, S.; Scott, L.; Phan, N.; Okwori, F.O.; Felemban, N.; Godebo, T.R. Toxic and Essential Elements in Rice and Other Grains from the United States and Other Countries. *Int. J. Environ. Res. Public Health* **2020**, *17*, 8128. https://doi.org/10.3390/IJERPH17218128.
41. Williams, P. N.; Raab, A.; Feldmann, J.; Meharg, A. A. Market Basket Survey Shows Elevated Levels of as in South Central U.S. Processed Rice Compared to California: Consequences for Human Dietary Exposure. *Environ. Sci. Technol.* 2007, *41* (7), 2178–2183. https://doi.org/10.1021/es061489k.

42. Zavala, Y. J.; Duxbury, J. M. Arsenic in Rice: I. Estimating Normal Levels of Total Arsenic in Rice Grain. *Environ. Sci. Technol.* 2008, *42* (10), 3856–3860. https://doi.org/10.1021/es702747y.

48. Kongsri, S.; Srinuttrakul, W.; Sola, P.; Busamongkol, A. Instrumental Neutron Activation Analysis of Selected Elements in Thai Jasmine Rice. *Energy Procedia* 2016, *89,* 361–365. https://doi.org/10.1016/j.egypro.2016.05.047.

49. Pinto, E.; Almeida, A.; Ferreira, I. M. P. L. V. O. Essential and Non-Essential/Toxic Elements in Rice Available in the Portuguese and Spanish Markets. *J. Food Compost. Anal.* 2016, *48*, 81–87. https://doi.org/10.1016/j.jfca.2016.02.008.

50. Rondan, F. S.; Hartwig, C. A.; Novo, D. L. R.; Moraes, D. P.; Cruz, S. M.; Mello, P. A.; Mesko, M. F. Ultra-Trace Determination of Bromine and Iodine in Rice by ICP-MS after Microwave-Induced Combustion. *J. Food Compost. Anal.* 2018, *66*, 199–204. https://doi.org/10.1016/j.jfca.2017.12.023.
